# The support of healthcare workers suffering from COVID 19

**DOI:** 10.1192/j.eurpsy.2022.1306

**Published:** 2022-09-01

**Authors:** I. Kacem, M. Maoua, A. Chouchane, M. Kahloul, Y. Slama, M. Ajmi, W. Naija, N. Mrizak

**Affiliations:** 1 Farhat Hached Academic Hospital, Occupational Medicine, Sousse, Tunisia; 2 Sahloul Academic Hospital, University of medicine, “Ibn Al Jazzar”, Sousse, Tunisia, Department Of Anesthesia And Intensive Care, Sousse, Tunisia

**Keywords:** support, healthcare workers, covid 19

## Abstract

**Introduction:**

The COVID-19 pandemic has focused attention on the challenges and risks faced by frontline healthcare workers (HCW).

**Objectives:**

To describe the quality of management of HCW affected by the COVID-19.

**Methods:**

This is a cross-sectional study enrolling all HCW of Farhat Hached Academic hospital who had been affected by COVID-19 during the period from september to December 2020.

**Results:**

During the study period, 267 HCW were affected with a mean age of 42.3 ±10 years and a ratio-sex of 0.25. The most represented category was nurses (33.3%) followed by technicians (26.1%). Gynecology department had the highest number of affected HCW (14.4%).The majority of participants (97.4%) reported a medical care. Twelve HCW (4.5%) were hospitalized with an average length of hospital stay of 7.55 ± 6.12 days. The average length of sick leave was 18.68 ± 10.99 days. During the lockdown, 38.6% of HCW took care of their children without any external help. All of the HCW were supported by phone calls from colleagues in 88.4% of cases, the hierarchy in 67.4% of cases, occupational medicine in 60.3% of cases.

**Conclusions:**

The impact of COVID 19 is greater in HCW than in the general population. The affected staff should have a multidimensional management to avoid post covid sequelae in both physical and mental levels.

**Disclosure:**

No significant relationships.

